# Trends in the Use of Conventional and New Pharmaceuticals for Hemophilia Treatments Among Medicaid Enrollees, 2005-2020

**DOI:** 10.1001/jamanetworkopen.2021.12044

**Published:** 2021-06-02

**Authors:** Inmaculada Hernandez, Deanna Rowe, Walid F Gellad, Chester B. Good

**Affiliations:** 1 Skaggs School of Pharmacy and Pharmaceutical Sciences, University of California at San Diego, La Jolla; 2 University of Pittsburgh Medical Clinic Health Plan, Insurance Services Division, Pittsburgh, Pennsylvania; 3 Division of General Internal Medicine, University of Pittsburgh School of Medicine, Pittsburgh, Pennsylvania

## Abstract

This cohort study used Medicaid data to examine trends in the use of and spending for hemophilia pharmaceuticals from 2005 to 2020.

## Introduction

Advances in the management of hemophilia have been accompanied by substantial increases in costs, making hemophilia one of the most expensive medical conditions to manage.^1^ Despite the high costs of hemophilia management, no contemporary peer-reviewed studies have examined trends in spending on hemophilia pharmaceuticals, particularly after extended half-life (EHL) factor and emicizumab entered the US market in 2014 and 2018, respectively. This is especially relevant to Medicaid, which provides insurance coverage for approximately half of all US patients with hemophilia.^2^ We leveraged Medicaid data to examine trends in the use and spending for hemophilia pharmaceuticals from 2005 through the second quarter of 2020.

## Methods

This was a cohort study for which institutional review board approval was waived because the study did not use human data and did not require informed patient consent. The Strengthening the Reporting of Observational Studies in Epidemiology (STROBE) reporting guideline was used.

Data for factor VIII and factor IX pharmaceuticals, bypassing agents, and emicizumab were extracted from reimbursement records from the first quarter of 2005 to the second quarter of 2020 in Medicaid state drug utilization data. Bypassing agents are used by patients with inhibitors, overcoming the need for factors blocked by inhibitors to continue the coagulation cascade. Emicizumab is a novel monoclonal antibody that bridges factors IXa and X, allowing the coagulation cascade to continue without factor VIII. We grouped factor pharmaceuticals according to their origin (plasma-derived vs recombinant) and half-life (standard vs extended). We estimated the number of units and the amount reimbursed by Medicaid for every quarter and pharmaceutical type. We calculated outpatient prerebate Medicaid spending on factor VIII, factor IX, and other agents per quarter and estimated the amount of spending accounted for by each pharmaceutical type. SAS statistical software version 9.4 (SAS Institute) was used for calculations; data analysis was performed from October 2020 to December 2020.

## Results

### Use

**Figure 1.**
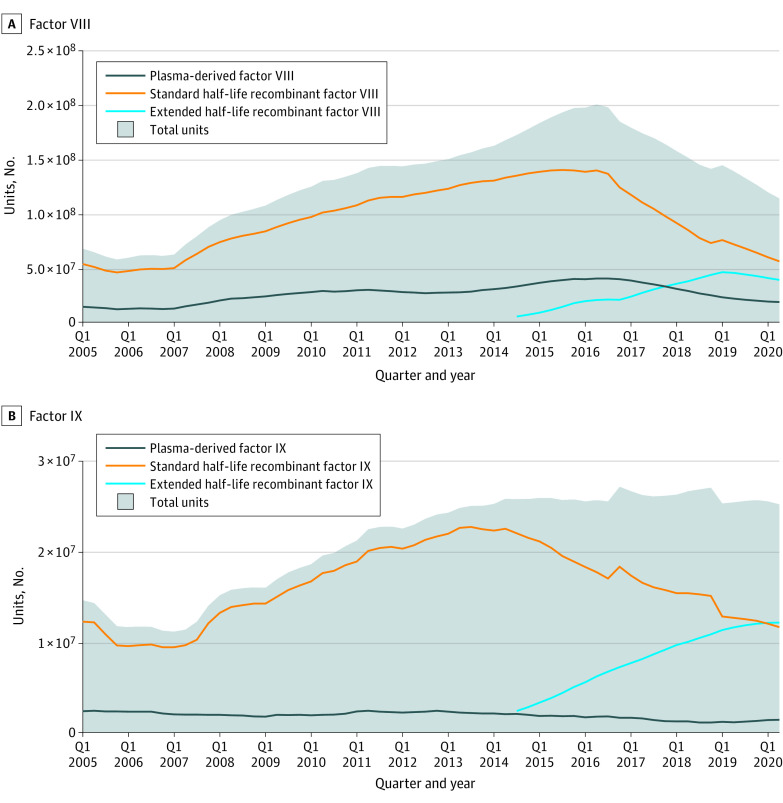
Number of Units of Hemophilia Pharmaceuticals Reimbursed by Medicaid per Quarter (Q), Q1 2005 to Q2 2020 The shadowed areas represent the total number of units reimbursed by Medicaid per Q for all factor VIII products (A) and all factor IX products (B). Plasma-derived factor VIII products included Hemofil, Monoclate, Alphanate, Humate, Koate, and Wilate. Standard half-life recombinant factor VIII products included Afstyla, Helixate, Kogenate, Novoeight, Nuwiq, Recombinate, Xyntha, Advate, and Kovaltry. Extended half-life recombinant factor VIII products included Adynovate, Eloctate, Esperoct, and Jivi. Plasma-derived factor IX products included Alphanine, Profilnine, and Mononine. Standard half-life recombinant factor IX products included Benefix, Ixinity, and Rixubis. Extended half-life recombinant factor IX products included Alprolix, Idelvion, and Rebinyn. Bypassing agents included Feiba and Novoseven.

Before 2015, there was a steady increase in the use of standard half-life (SHL) recombinant factor, which dominated the market (Figure 1). After the entry of EHL recombinant factor in the third quarter of 2014, the use of SHL factor decreased from 142 087 494 units to 48 161 021 units (a 66% decrease) for factor VIII and from 24 724 386 units to 10 682 603 units (a 57% decrease) for factor IX. After the entry of EHL factor in the US market, total units decreased from 175 019 342 units to 103 771 307 units (a 41% decrease) for factor VIII but remained constant for factor IX. From 2017 to 2019, the use of bypassing agents decreased from 11 634 758 units to 3 558 170 units (a 69% decrease).

### Spending

**Figure 2.**
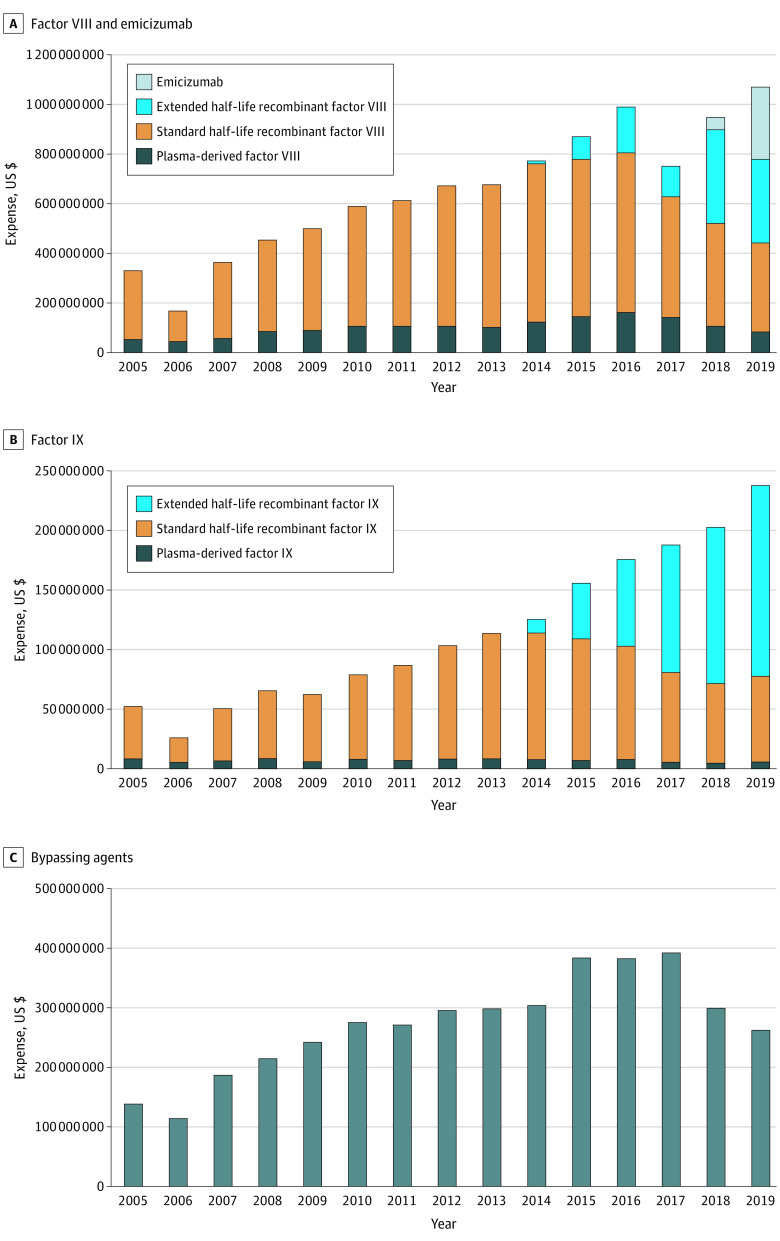
Annual Medicaid Spending for Hemophilia Products, 2005-2019

Total Medicaid spending in hemophilia pharmaceuticals tripled from 2005 to 2019, from $521 million in 2005 to $1.57 billion in 2019. Across the study period, annual spending more than doubled for factor VIII (from $330 million in 2005 to $779 million in 2019) and more than quadrupled for factor IX (from $52 million in 2005 to $238 million in 2019) (Figure 2). In 2019, only 1 year after emicizumab approval, factor VIII represented $778 592 692 of $1 569 746 508 (50%) of total Medicaid spending on hemophilia pharmaceuticals; factor IX, $237 846 173 (15%); bypassing agents, $262 242 517 (17%); and emicizumab, $291 065 124 (19%).

## Discussion

The transition of factors VIII and IX from plasma-derived to recombinant pharmaceuticals, the transition from SHL to EHL pharmaceuticals, and the use of bypassing agents and emicizumab have contributed to the transformation of hemophilia from a disease of significant morbidity to a condition that allows affected individuals to lead active lives. However, these advances have increased costs substantially.^3^ From 2005 to 2020, Medicaid spending on hemophilia pharmaceuticals tripled, increasing by more than $1 billion.

Use of factor VIII decreased with the availability of EHL recombinant pharmaceuticals and emicizumab, and unlike hemophilia A, the entry of EHL recombinant pharmaceuticals did not mitigate the dosing of factor IX. Furthermore, no significant change in the use of factor VIII was observed after the publication of the Survey of Inhibitors in Plasma-Product Exposed Toddlers data, which demonstrated that patients treated with plasma-derived factor VIII had a lower incidence of inhibitors than those treated with recombinant factor.^4^

This study was limited because the lack of patient data precluded patient-level analyses, which would have been necessary to understand the underlying patterns behind the observed change in the use of each pharmaceutical. The unavailability of discount data prevented us from estimating net spending on hemophilia pharmaceuticals.

Our findings are relevant to state Medicaid budgets, particularly because state agencies face coverage decisions after the expected approval of multiple gene therapies for hemophilia in the next decade. These gene therapies, which will cost between $2 to $3 million per patient,^2^ have the potential to decrease requirements for factor replacement dramatically.^5^
